# Waste in orthopaedic surgery; an application of the healthcare sustainability mode and effect analysis

**DOI:** 10.1007/s00264-025-06629-7

**Published:** 2025-08-08

**Authors:** Isabella C. Klarenbeek, Esther R.C. Janssen, Paul C. Willems, Okke F. Lambers Heerspink, Anne C. van der Eijk

**Affiliations:** 1 Department of Orthopaedic Surgery, Viecuri Medisch Centrum, Venlo, Netherlands; 2 Department of Orthopaedics and Research School Caphri, Maastricht University, Maastricht, Netherlands; 3 IQ health, Radboud University Medical Center, Radboud Institute for Health Sciences, Nijmegen, Netherlands; 4 School of Allied Health, HAN University of Applied Sciences, Nijmegen, Netherlands; 5 Operating Room Department and Central Sterile Supply Department, Leiden University Medical Center, Leiden, Netherlands; 6 Department of Biomedical Engineering, Delft University of Technology, Delft, Netherlands

**Keywords:** Arthroplasty, Orthopaedic, Waste assessment, Hospital waste reduction, Planetary health, Environmental sustainability, Life Cycle Assessment, Waste analysis, Operation room

## Abstract

**Introduction:**

Operating theatres generate a lot of waste and as such are a major contributor to the rise of negative environmental impact of hospitals. Thus reducing operating room waste is an essential strategy for hospitals to reduce their environmental impact and contribute to a healthier environment. This study aims to quantify waste from six common orthopaedic procedures and identify potential strategies for reduction.

**Methods:**

The Healthcare Sustainability Mode and Effect Analysis (HSMEA) is a method to assess the environmental impact of waste. It is a systematic approach to analyse waste, calculate the environmental impact and identify strategies to reduce this impact. In this study an HSMEA of operating room waste of 18 orthopaedic procedures was performed: open and percutaneous spinal fusion (*n* = 6), unicompartmental and total knee arthroplasty (*n* = 6), reverse shoulder arthroplasty (*n* = 3) and total hip arthroplasty (*n* = 3). For each type of waste, the strategies of the 6R methodology were considered to reduce the environmental impact of the operating theatre department.

**Results:**

The weight of the waste of orthopaedic procedures ranged between 6.35 and 8.30 kg. About 70% of the total waste was plastic. The environmental impact of measured orthopaedic procedures ranged between 19.14 and 23.96 kg CO_2_-eq. The impact of the six orthopaedic procedures could be reduced with 10.3 to 13.9 kg CO_2_-eq. using the 6R methodology.

**Conclusion:**

The environmental impact of waste from orthopaedic procedures is substantial and can be reduced up to 63%. Applying the HSMEA method to all procedures in the operating theatres can lead to a significant reduction of the carbon footprint of hospitals.

**Supplementary Information:**

The online version contains supplementary material available at 10.1007/s00264-025-06629-7.

## Introduction

The global carbon footprint of healthcare is approximately 5% in countries with market-based economies [1]. This presents a paradox: while the fundamental purpose of healthcare is to improve human health, the associated greenhouse gas emissions undermine this goal [1–4]. The operating theatre department has the largest carbon footprint per square metre of the hospital and is the department generating the most waste (20–30% of total hospital waste) [2–6]. The amount of waste varies substantially between procedures, whilst existing research is limited to a few specific procedures. For most procedures, neither the weight nor the environmental impact of the waste has been studied before [7–10].

Orthopaedic surgery is among the specialties producing the most surgical waste associated with the surgical procedure itself [11]. Despite this, research on the quantity and environmental impact of waste from orthopaedic procedures is limited and urgently needed [11, 12]. Only a few orthopaedic procedures have been studied, including total knee arthroplasty (TKA) and total hip arthroplasty (THA). These procedures produce between 11.6 and 15.1 kg of waste [13–18]. Only two of these studies reported the environmental impact of the waste [17, 19]. Delaine et al. [19] reported that the life cycle environmental impact of the whole TKA procedure was 190.5 kg of CO_2_-eq. Drouaud et al. [17] reported that the environmental impact of the generated waste from TKA was 15.82 kg CO_2_-eq. and THA was 14.64 kg CO_2_-eq. This study aims to be the first study to perform a waste audit study on a broad range of common orthopaedic procedures and identify the possible strategies to reduce orthopaedic waste using the Healthcare Sustainability Mode and Effect Analysis (HSMEA).

## Methods

### Study design

This study was a single-centre observational qualitative study, performed in a public non-academic teaching hospital in the Netherlands. In our hospital’s operating theatre department, more than 2000 orthopaedic procedures are performed each year. Surgical instruments are packaged in instrument trays using disposable polypropylene wrap.

This study was reported according to the STROBE & SQUIRE 2.0 guideline (Supplemental file A). The study was approved by the local ethics committee (no. 2024-0082). Informed consent was obtained from all patients. All data was collected between December, 2023 and May, 2024.

### Healthcare sustainability mode and effect analysis

**Fig. 1:**
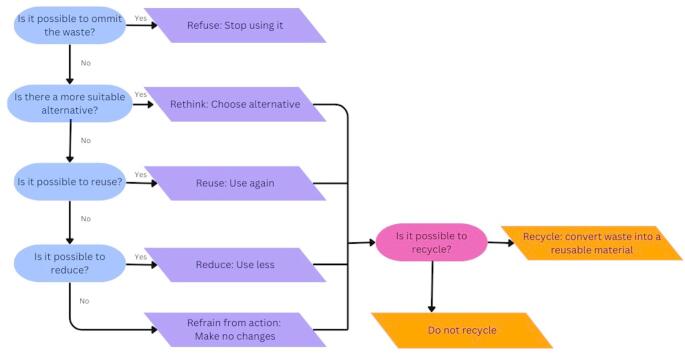
Decision tree used in step 4 hazard analysis. The adjusted R ladder is based on the Ridder et al., 2022 and Kagoma et al., 2012. It includes the 6R methodology: refuse, rethink, reuse, reduce, refrain from action and recycle

The HSMEA is a method used to asses healthcare waste [20]. The method’s reproducible and structured approach offers a framework for identifying carbon hotspots and executing solutions to reduce the associated carbon footprint. The HSMEA follows a meticulously structured, step-by-step process [20, 21]. The five steps of an HSMEA are: ‘definition of the topic’, ‘team assembly’, ‘flowchart creation’, ‘hazard analysis’, and ‘actions and outcome measures’ (Supplemental file B). During the hazard analysis, the 6R methodology was applied. These 6R’s are: ‘Refuse’, ‘Rethink’, ‘Reuse’, ‘Reduce’, ‘Refrain from action’ and ‘Recycle’, see Fig. 1.

In our study we included the following procedures: ‘open spinal fusion’ (max. three levels) (n = 3), ‘percutaneous spinal fusion’ (max. three levels)(n = 3), ‘unicompartmental knee arthroplasty’ (cemented UKA)(n = 3), ‘total knee arthroplasty’ (cemented TKA)(n = 3), ‘reverse shoulder arthroplasty’ (uncemented RSA)(n = 3) and’ total hip arthroplasty’ (uncemented THA)(*n* = 3). All procedures were primary elective surgeries in adults with a degenerative disorder. Given the high level of standardisation in these procedures and expected minimal variation within each type of surgery, waste was collected from three patients per procedure.

### Calculation

In the HSMEA each identified sub-step in the flowchart received an environmental hazard score [1]. The environmental impact was calculated by multiplying the waste weight (kg) with conversion factors (kg CO_2_ equivalents). A detailed explanation on the conversion factors can be found in supplemental file B. All data were analysed using Excel (Microsoft office 2016). To reduce the environmental impact, the different strategies of the 6R methodology were considered by the multidisciplinary team. The most sustainable strategy was chosen, provided its implementation was feasible in our hospital (Table 1, appendix B).

## Results: hazard analysis

**Fig. 2:**
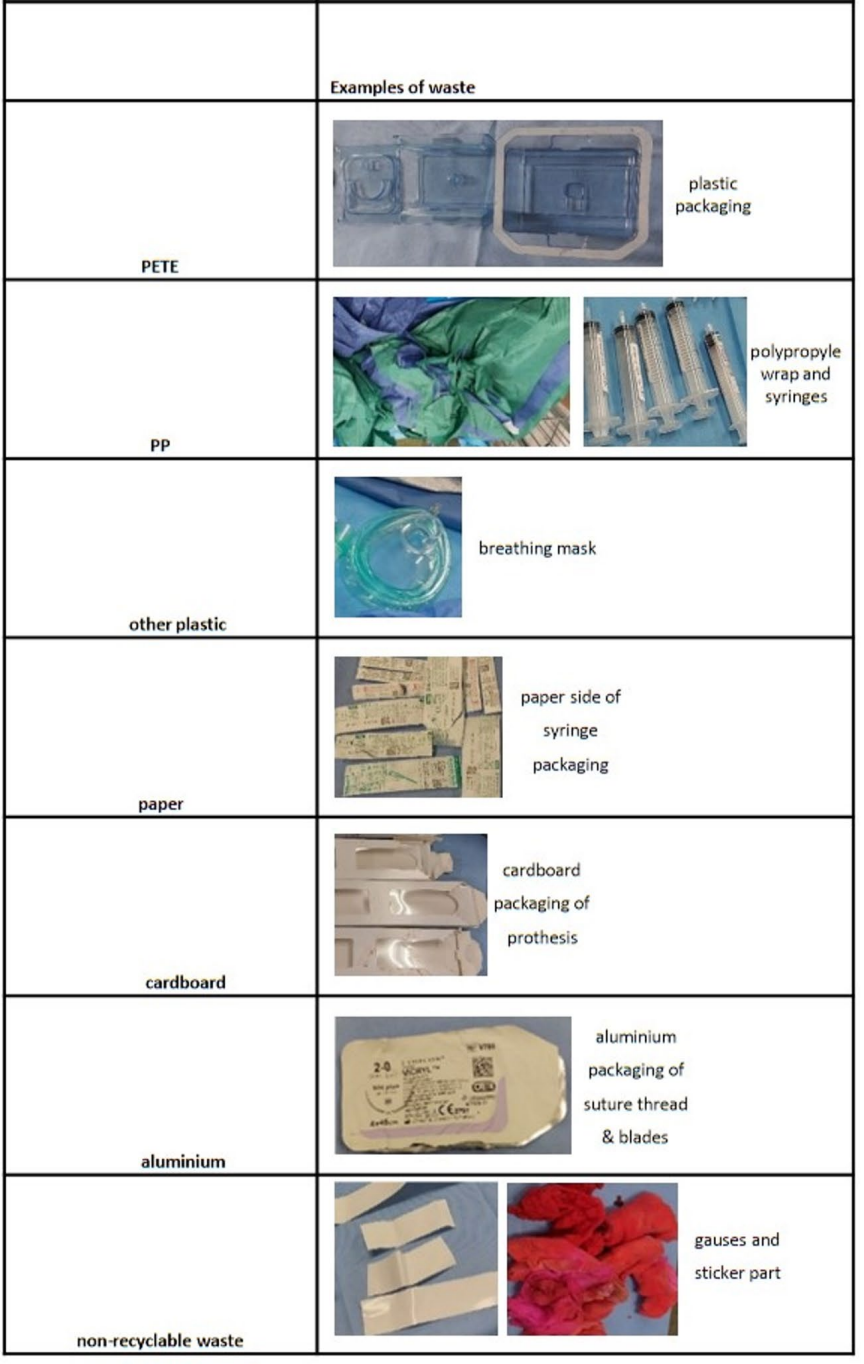
Overview of the different waste production categories the waste was divided into

The surgical waste of 18 procedures was analysed and divided into seven waste categories, see Fig. 2. The mean age of included patients was 72.0 (SD = 12.4). The weight of the waste from the orthopaedic procedures ranged between 6.05 and 8.80 kg (Table 1).

**Fig. 3:**
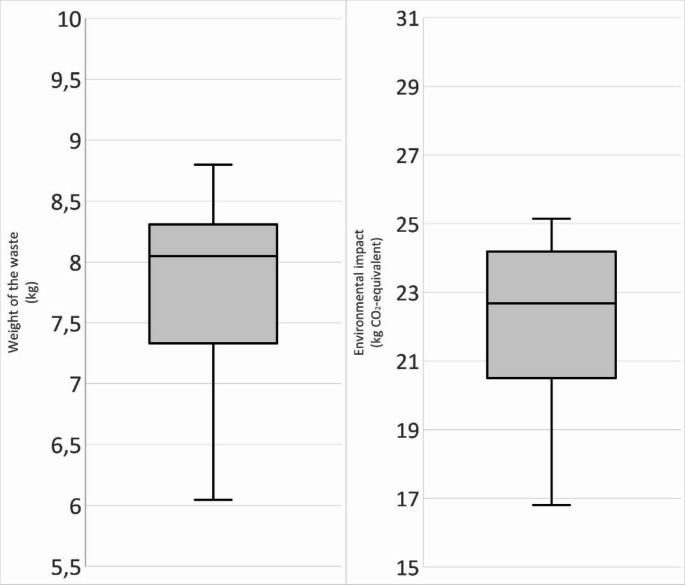
Boxplot of the weight of the waste (*n* = 18) and the environmental impact (CO_2_-equivalent). The median of the weight was 7.93 kg and of the environmental impact was 22.68 kg CO_2_-eq. The minimum of the weight was 6.05 and the maximum was 8.80 kg. The minimum of the environmental impact was 16.81 and the maximum was 25.14 kg CO_2_-eq

The mean plastic waste (PETE + PP + other plastics) for the different types of procedures was 5.26 kg (sd = 0.801 kg) and accounted for 69% of the total waste weight. The waste category with the highest weight was PP ranging between 2.67 and 4.80 kg per procedure (*n* = 18). RSA (3.79–4.80 kg) (*n* = 3) generated the most PP waste, while UKA (2.797–3.36 kg) produced the least amount PP (*n* = 3), see Table 1. The median of the weight of all the orthopaedic procedures was 7.93 kg (Fig. 3).

**Table 1:** Overview of the mean weight of different waste production across procedures and the total weight of the waste per procedure (in grams). The waste weight (kg) was multiplied with the conversion factors (kg CO2 equivalents). A detailed explanation is available in appendix B

	PETE (grams)	PP(grams)	other plastic(grams)	paper(grams)	cardboard(grams)	Aluminium (grams)	non-recyclable waste (grams)	total(grams)	
THA (*n* = 3)	272	3 467	1 153	166	354	9	928	6 349	
Open Spinal Fusion (*n* = 3)	327	3 947	1 401	355	396	3	1 868	8 297	
Percutaneous Spinal Fusion (*n* = 3)	297	4 026	1 401	320	352	7	1 541	7 944	
TKA (*n* = 3)	604	3 349	1 192	232	279	12	2 348	8 017	
RSA (*n* = 3)	342	4 286	1 218	263	338	9	1 543	8 000	
UKA (*n* = 3)	243	3 149	9 12	422	272	9	2 374	7 382	

THA = total hip arthroplasty, TKA = total knee arthroplasty, RSA = reverse shoulder arthroplasty, UKA = unicompartmental knee arthroplasty PETE = polyethylene terephthalate PP = polypropylene plastic *=* high-density polyethylene (HDPE), polyvinyl chloride (PVC), low-density polyethylene (LDPE) & polystyrene (PS)

The mean of the environmental impact of the measured orthopaedic procedures ranged between 19.14 and 23.96 kg CO_2_-eq. (Table 2, *n* = 18). The median of the environmental impact of all the procedures was 22.68 kg CO_2_-eq. (Fig. 3, *n* = 18). Open spinal fusion had the highest environmental impact, which ranged between 23.43 and 24.29 kg CO_2_-eq. (*n* = 3), while THA had the lowest impact 16.81–20.70 kg CO_2_-eq. (*n* = 3).

**Table 2:** Environmental impact of orthopaedic waste as expressed in mean CO2-eq. per procedure before (A), and after adding recycling (B). All categories, except cardboard, were incinerated. The environmental impact of cardboard was based on recycling. After adding recycling, plastic and aluminium were recycled instead of incinerated. The waste weight (kg) was multiplied with the conversion factors (kg CO2 equivalents). A detailed explanation is available in appendix B

**A. Environmental impact of orthopaedic waste per procedure**								
	**PETE**	**PP**	**other plastic**	**paper**	**cardboard**	**aluminum**	**non-recyclable waste**	**total**
THA (*n* = 3)	1 179	9 904	5 774	34	214	92	1940	19,138
Open Spinal Fusion (*n* = 3)	1 420	11 277	7 015	73	239	31	3905	23,959
Percutaneous Spinal Fusion (*n* = 3)	1 291	11 501	7 014	66	212	70	3221	23,376
TKA (*n* = 3)	2 621	9 569	5 972	48	168	119	4908	23,404
RSA (*n* = 3)	1 485	12 246	6 102	54	204	85	3225	23,402
UKA (*n* = 3)	1 057	8 997	4 567	87	164	92	4961	19,925
**B. Environmental impact of orthopaedic waste per procedure if plastic and aluminium are recycled instead of incinerated**								
	**PETE**	**PP**	**other plastic**	**paper**	**cardboard**	**aluminum**	**non-recyclable waste**	**total**
THA (*n* = 3)	306	6 503	3 700	-67	214	6	1 940	12 602
Open Spinal Fusion (*n* = 3)	368	7 405	4 495	-143	239	2	3 905	16 270
Percutaneous Spinal Fusion (*n* = 3)	334	7 552	4 494	-129	212	4	3 221	15 689
TKA (*n* = 3)	679	6 283	3 827	-94	168	7	4 908	15 778
RSA (*n* = 3)	385	8 041	3 910	-106	204	5	3 225	15 664
UKA (*n* = 3)	274	5 908	2 926	-170	164	6	4 961	14 069

THA = total hip arthroplasty, TKA = total knee arthroplasty, RSA = reverse shoulder arthroplasty, UKA = unicompartmental knee arthroplasty PETE = polyethylene terephthalate PP = polypropylene

## Results: actions and outcome measures

### Recycling

**Fig. 4:**
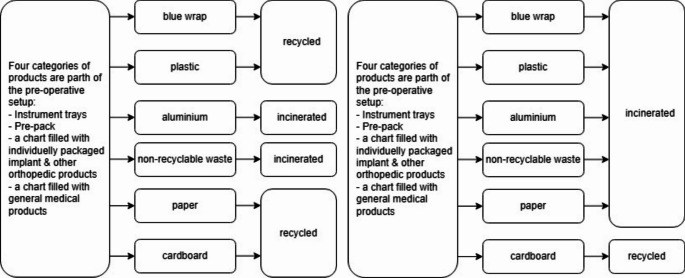
Process flow chart of the process before and after changes

Cardboard is already recycled in our hospital, while materials such as ‘other plastics’ (including PETE and PP), paper, and aluminium are not recycled (Fig. 4). We aim to make the entire surgical waste stream recyclable. The environmental impact of the waste generated from the measured orthopaedic procedures could be reduced by 7.19 kg CO_2_-eq. (Table 2, n = 18) by recycling plastic, paper and aluminium.

### Refuse

Our institution uses procedural packs for RSA, UKA, TKA and THA, which are pre-assembled packages of sterile medical supplies. UKA and TKA use the same procedural pack. These procedural packs contain surgical gowns, tubes, sheets, cloths, gauzes, bandages, diathermy bags, and surgical gloves. The content of the these were evaluated using the 6R methodology. We were able to remove paper towels (+ 1 g CO_2_-eq.), covering material for the table (-406 g CO_2_-eq.) and syringes (-71 g CO_2_-eq.) from the RSA procedural pack as they were not routinely used. For the knee arthroplasty procedural pack, we were able to remove paper towels (+ 1 g CO_2_-eq.), an adhesive strip (+ 28 g CO_2_-eq.) and a syringe (-115 g CO_2_-eq.). These items were unused and thrown away during the procedure. An overview of the removed items from the procedural packs and procedure set-up list is available in the supplemental file B.

For THA, we were able to remove two instruction manuals, in cooperation with the manufacturer, from the implant hip packages, which were replaced with QR codes (-15 g CO_2_-eq.). Bone cement was part of the standard setup and used during all UKA procedures in our hospital, including the three UKA procedures of which the waste was analysed. We switched the UKA procedures from being standard cemented to uncemented, so we were able to remove the bone cement, the craft kit, its packaging and the pulse lavage from our waste (-2.624 g CO_2_-eq.).

### Rethink

In total 58 disposable surgical gowns were used across all 18 procedures, averaging 3.22 gowns per procedure. We will replace these with reusable gowns (removing all the gowns from the waste). Each procedure used large PP patient cover material. For the RSA (-539 g CO_2_-eq.) and THA (-707 CO_2_-eq.) we were able to change from three layers PP to two layers, as our patient temperature management system and an additional blanket already provided sufficient warmth.

### Overall reduction operating room waste

An overview of potential and implemented changes can be found in Table 2. The strategy ‘refrain from action’ was adopted for all other waste. The changes could result in a reduction of the weight of the waste between 5.80 kg (open spinal fusion) and 17.07 kg (UKA) depending on the procedure (Table 3). The environmental impact (CO₂-eq.) related to this waste would be reduced between 10.71 kg (THA) and 14.27 kg (RSA), depending on the procedure (Table 3). Resulting in a reduction of 10.34–13.89 kg CO₂-eq. per procedure when all 6R strategies are implemented.

**Table 3:** Reduction of the environmental impact related the waste that is thrown away during a procedure, by applying the 6R methodology. The waste weight (kg) was multiplied with the conversion factors (kg CO2 equivalents). A detailed explanation is available in appendix B

	Mean waste weight (grams)	Reduction of the waste weight (grams)	Reduction of the waste weight (%)	Mean g CO_2_-eq. prodcued	Reduction of g CO2-eq. because of recycling	Reduction of g CO2-eq. related to the waste by using Refuse, Rethink, Reuse, Reduce	Reduction of the overall CO2-eq. of waste	Reduction of environmental impact (%)
THA (3)	6 349	1 020	16%	19 138	6 537	4 173	10 710	56%
open spinal fusion (3)	8 297	580	7%	23 959	7 690	5 395	13 084	55%
percutaneous spinal fusion (3)	7 944	707	9%	23 376	7 686	5 535	13 221	57%
TKA (3)	8 017	600	7%	23 404	7 626	3 599	11 225	48%
RSA (3)	8 000	1 262	16%	23 402	7 738	6 528	14 266	61%
UKA (3)	7 382	1 707	23%	19 925	5 857	6 752	12 608	63%

## Discussion

In this study the waste from six common orthopaedic procedures and its associated carbon footprint was quantified, and possible strategies for waste reduction were identified. The mean weight of the waste generated by the included orthopaedic procedures was 7.66 kg and the mean environmental impact was 22.20 kg CO_2_-eq. Waste is one of the areas over which hospitals have significant influence to reduce the environmental impact, as was shown in our study by the observed 10.34–13.89 kg CO_2_-Eqs. (48–63%) reduction.

Several studies have explored the waste generated during TKA and THA [13–19, 22, 23]. Common strategies to reduce the environmental impact include increase of recycling [13–19], replacing disposables with reusables [13, 19], precision-based technologies to minimise the over-selection of implants [22, 23], and updating the procedure trays regularly [17]. Our findings align with these strategies. However, the reported potential for waste reduction across studies varied significantly. This variability can be attributed to differences in the inclusion and exclusion criteria of categories of waste. For example, some studies included clinical hazardous waste (for example blood) in their assessments [13, 14], which contributed to higher reported reduction outcomes. Differences in surgical technique, instruments, and materials used across studies may also explain this variability.

Our findings highlight the possibility to reduce waste using waste reduction strategies in hospitals, as it represents an effective area for improving the environmental sustainability of orthopaedic procedures that can directly be implemented. This represents a practical and effective opportunity to enhance the environmental sustainability of orthopaedic procedures.

### Strengths and limitations

A strength of the HSMEA method is that it can effeciently reduce environmental impact and aligns well with the aim of this study, which was to quantify waste from orthopaedic procedures and identify potential strategies for its reduction. The HSMEA has the advantage of allowing for quick identification and direct implementation of sustainable changes in practice. To the best of our knowledge this study was to first study to perform a waste audit using the validated HSMEA method and to implement the changes in clinical practice.

An example of a change we implemented was switching from cemented UKA to uncemented UKA, based on our findings that removing certain items from our procedural pack would lower the environmental impact of the waste (-2.624 g CO2-eq.). However, generating less waste does not necessarily equate to a lower overall environmental impact. While our results showed that uncemented UKA generated less waste, this comparison did not account for other contributing factors. In only three articles it was addressed whether cemented or uncemented implants were used and none directly compared the waste generated by each type [13, 18, 19]. A complete LCA that accounts for the production of both implant types would be necessary to accurately evaluate their environmental footprints in detail. It is also important to note that the eventual choice between implant types should be guided primarily by clinical judgment and patient-specific factors. Sustainability should be considered when both options are clinically equivalent.

This study was limited to six common orthopaedic procedures. These procedures were selected with the expectation that they would offer significant potential for waste reduction. While this study quantifies the waste generated by these procedures, it does not provide a comprehensive assessment of waste production across all orthopaedic procedures. We expect that orthopaedic procedures not included, would produce a similar or smaller amounts of waste. Furthermore, techniques, instruments and materials vary between hospitals. Due to variability in organisation between hospitals, achieving the same level of waste reduction may not be feasible at every centre. While our results account specifically for our hospital, they highlight the potential for reducing carbon dioxide emissions in orthopaedic surgery. This study offers a method that can be used in all centres to monitor and reduce the weight and environmental impact of the waste of orthopaedic and other procedures.

We found minimal variation in the items used across procedures, which can be explained by the high standardization of arthroplasty procedures. Differences in weight of the waste within the same procedure were primarily due to differences in bloody gauzes, surgical gloves, and the protective equipment worn by surgical observers (residents and students), which can differ regardless of procedure type. Given our aim to identify strategies to reduce the environmental impact of six common highly standardized orthopaedic procedures, we believe the number of procedures analysed to be sufficient.

To calculate the environmental impact, an upcycling credit was applied. A positive credit (+) was used to account for environmental savings, even though the actual recycling and incineration of waste occurred ouside our direct supply chain. This approach allowed us to highlight the difference in environmental impact between recycling and incineration.

### Clinical implications

This study, along with previous research, highlights the possibility and need to reduce waste in orthopaedic procedures. We found that some items were included in the procedure setup out of routine rather than necessity. Promoting a culture of sustainability is crucial, as operating room staff are well placed to identifyand eliminate unnecessary waste.

Efforts should focus on avoiding unnecessary materials, such as surgical instrument sets, since their packaging of sets contributes to waste [24–26]. Wherever possible, disposable items should be replaced with reusables. Ultimately, the most sustainable approach to healthcare is to reduce unnecessary operative care whenever possible.

Hospitals should adopt and implement comprehensive sustainable waste management strategies across their operations. These strategies include refusing unnecessary items, reassessing the need for certain materials, reusing when safe, reducing overall volume, avoiding single-use items when alternatives exist, and actively recycling. The strategies should be applied regularly, as new research and innovations in sustainable alternatives continue to emerge. To effectively drive sustainability, hospitals should collaborate closely with their staff, industry partners, institutions, and government agencies. By fostering these partnerships and embracing ongoing change, hospitals can continally reduce their environmental footprint.

## Conclusion

Implementing sustainable strategies based on the 6R methodology can significantly reduce the environmental impact of orthopaedic procedures. Waste reduction is one of the most actionable areas for improvement for hospitals. Hospitals should foster a culture of sustainability in the operating theatre, ensuring that unnecessary materials are eliminated, reusable alternatives are prioritised, and waste management strategies are consistently applied. Even small changes, when scaled across procedures, can lead to substantial environmental benefits.

## Supplementary Information

Below is the link to the electronic supplementary material.

Supplementary Material 1

Supplementary Material 2

## Data Availability

No datasets were generated or analysed during the current study.
