# Supplementary material for: Waste in orthopaedic surgery; an application of the healthcare sustainability mode and effect analysis
Source: Int Orthop. 2025 Aug 8;49(10):2393–401. doi: 10.1007/s00264-025-06629-7 (PMC12488813; doi:10.1007/s00264-025-06629-7)
Supplement: Supplementary file 1 — Supplementary Material 1 [file 264_2025_6629_MOESM1_ESM.docx]

# Supplemental file A

**Table 1**. Square checklist 2.0 STROBE Statement—checklist of items that should be included in reports of observational studies (1). In accordance with the SQUIRE guidelines, all items were considered. Items deemed inappropriate or unnecessary for the study design were marked as not applicable.

|  | Item No | Recommendation | Page  No |
| --- | --- | --- | --- |
| **Title and abstract** | 1 | (*a*) Indicate the study’s design with a commonly used term in the title or the abstract | Page 1 |
|  |  | (*b*) Provide in the abstract an informative and balanced summary of what was done and what was found | Page 2-3 |
| Introduction | | | |
| Background/rationale | 2 | Explain the scientific background and rationale for the investigation being reported | Page 4 |
| Objectives | 3 | State specific objectives, including any prespecified hypotheses | Page 4 |
| Methods | | | |
| Study design | 4 | Present key elements of study design early in the paper | Page 5-7 |
| Setting | 5 | Describe the setting, locations, and relevant dates, including periods of recruitment, exposure, follow-up, and data collection | Page 5-7 |
| Participants | 6 | (*a*) *Cohort study*—Give the eligibility criteria, and the sources and methods of selection of participants. Describe methods of follow-up  *Case-control study*—Give the eligibility criteria, and the sources and methods of case ascertainment and control selection. Give the rationale for the choice of cases and controls  *Cross-sectional study*—Give the eligibility criteria, and the sources and methods of selection of participants | Page 5 |
|  |  | (*b*) *Cohort study*—For matched studies, give matching criteria and number of exposed and unexposed  *Case-control study*—For matched studies, give matching criteria and the number of controls per case |  |
| Variables | 7 | Clearly define all outcomes, exposures, predictors, potential confounders, and effect modifiers. Give diagnostic criteria, if applicable | Supplemental file B |
| Data sources/ measurement | 8* | For each variable of interest, give sources of data and details of methods of assessment (measurement). Describe comparability of assessment methods if there is more than one group |  |
| Bias | 9 | Describe any efforts to address potential sources of bias | Supplemental file B |
| Study size | 10 | Explain how the study size was arrived at | Supplemental file B |
| Quantitative variables | 11 | Explain how quantitative variables were handled in the analyses. If applicable, describe which groupings were chosen and why | Supplemental file B |
| Statistical methods | 12 | (*a*) Describe all statistical methods, including those used to control for confounding | Supplemental file B |
|  |  | (*b*) Describe any methods used to examine subgroups and interactions | Supplemental file B |
|  |  | (*c*) Explain how missing data were addressed | Not applicable |
|  |  | (*d*) *Cohort study*—If applicable, explain how loss to follow-up was addressed  *Case-control study*—If applicable, explain how matching of cases and controls was addressed  *Cross-sectional study*—If applicable, describe analytical methods taking account of sampling strategy | Not applicable |
|  |  | (*e*) Describe any sensitivity analyses |  |

| Results | | | |
| --- | --- | --- | --- |
| Participants | 13* | (a) Report numbers of individuals at each stage of study—eg numbers potentially eligible, examined for eligibility, confirmed eligible, included in the study, completing follow-up, and analysed | Page 7 |
|  |  | (b) Give reasons for non-participation at each stage | Not applicable |
|  |  | (c) Consider use of a flow diagram | Figure 1 |
| Descriptive data | 14* | (a) Give characteristics of study participants (eg demographic, clinical, social) and information on exposures and potential confounders | Supplemental file B |
|  |  | (b) Indicate number of participants with missing data for each variable of interest | Not applicable |
|  |  | (c) *Cohort study*—Summarise follow-up time (eg, average and total amount) |  |
| Outcome data | 15* | *Cohort study*—Report numbers of outcome events or summary measures over time |  |
|  |  | *Case-control study—*Report numbers in each exposure category, or summary measures of exposure |  |
|  |  | *Cross-sectional study—*Report numbers of outcome events or summary measures |  |
| Main results | 16 | (*a*) Give unadjusted estimates and, if applicable, confounder-adjusted estimates and their precision (eg, 95% confidence interval). Make clear which confounders were adjusted for and why they were included | Page 7-14 |
|  |  | (*b*) Report category boundaries when continuous variables were categorized | Page 7-14 |
|  |  | (*c*) If relevant, consider translating estimates of relative risk into absolute risk for a meaningful time period | Not applicable |
| Other analyses | 17 | Report other analyses done—eg analyses of subgroups and interactions, and sensitivity analyses | Page 8-14 |
| Discussion | | | |
| Key results | 18 | Summarise key results with reference to study objectives | Page 15 |
| Limitations | 19 | Discuss limitations of the study, taking into account sources of potential bias or imprecision. Discuss both direction and magnitude of any potential bias | Page 15-18 |
| Interpretation | 20 | Give a cautious overall interpretation of results considering objectives, limitations, multiplicity of analyses, results from similar studies, and other relevant evidence | Page 15-18 |
| Generalisability | 21 | Discuss the generalisability (external validity) of the study results | Page 17 - 18 |
| Other information | | | |
| Funding | 22 | Give the source of funding and the role of the funders for the present study and, if applicable, for the original study on which the present article is based | Not applicable |

**Table 2**. Square checklist 2.0 (2, 3).

|  |  | |  |
| --- | --- | --- | --- |
| **Text section and item name** | | **Info is located** | |
| **Title and abstract** | |  | |
| 1. Title | | Page 1 | |
| 2. Abstract | | Page 2-3 | |
| **Introduction: Why did you start?** | |  | |
| 3. Problem description | | Page 4 | |
| 4. Available knowledge | | Page 4 | |
| 5. Rationale | | Page 4 | |
| 6. Specific aims | | Page 4 | |
| **Methods: What did you do?** | |  | |
| 7. Context - | | Page 5-7 | |
| 8. Intervention(s) | |  | |
| a. Description of the intervention(s) in sufficient detail that others could reproduce it. | | Supplemental file B | |
| b. Specifics of the team involved in the work. | | Supplemental file B | |
| 9. Study of the intervention(s) | |  | |
| a. Approach chosen for assessing the impact of the intervention(s). | | Supplemental file B | |
| b. Approach used to establish whether the observed outcomes were due to the intervention(s). | | Not applicable | |
| 10. Measures | |  | |
| a. Measures chosen for studying processes and outcomes of the intervention(s), including rationale for choosing them, their operational definitions and their validity and reliability. | | Supplemental file B | |
| b. Description of the approach to the ongoing assessment of contextual elements that contributed to the success, failure, efficiency and cost. | | Not applicable | |
| c. Methods employed for assessing completeness and accuracy of data. | | Supplemental file B | |
| 11. Analysis | |  | |
| a. Qualitative and quantitative methods used to draw inferences from the data. | | Not applicable | |
| b. Methods for understanding variation within the data, including the effects of time as a variable. | | Not applicable | |
| 12. Ethical considerations | | Page 5 | |
| **Results: What did you find?** | |  | |
| 13. Results | |  | |
| a. Initial steps of the intervention(s) and their evolution over time (eg, time-line diagram, flow chart or table), including modifications made to the intervention during the project. | | Page 7-14 | |
| b. Details of the process measures and outcomes. | | Page 7-14 | |
| c. Contextual elements that interacted with the intervention(s). | | Page 14 | |
| d. Observed associations between outcomes, interventions and relevant contextual elements. | | Page 14 | |
| e. Unintended consequences such as unexpected benefits, problems, failures or costs associated with the intervention(s). | | Not applicable | |
| f. Details about missing data. | | Not applicable | |
| **Discussion: What does it mean?** | |  | |
| 14. Summary | |  | |
| a. Key findings, including relevance to the rationale and specific aims. | | Page 15 | |
| b. Particular strengths of the project. | | Page 16-17 | |
| 15. Interpretation | |  | |
| a. Nature of the association between the intervention(s) and the outcomes. | | Page 15 | |
| b. Comparison of results with findings from other publications. | | Page 15-17 | |
| c. Impact of the project on people and systems. | | Page 15-18 | |
| d. Reasons for any differences between observed and anticipated outcomes, including the influence of context. | | Not applicable | |
| e. Costs and strategic trade-offs, including opportunity costs. | | Not applicable | |
| 16. Limitations | |  | |
| a. Limits to the generalisability of the work. | | Page 17 | |
| b. Factors that might have limited internal validity such as confounding, bias or imprecision in the design, methods, measurement or analysis. | | Not applicable | |
| c. Efforts made to minimise and adjust for limitations. | | Not applicable | |
| **Conclusions** | |  | |
| a. Usefulness of the work. | | Page 19 | |
| b. Sustainability. | | Page 17-19 | |
| c. Potential for spread to other contexts. | | Page 17-18 | |
| d. Implications for practice and for further study in the field. | | Page 16-18 | |
| e. Suggested next steps. | | Page 17-18 | |
| 18. Funding - Sources of funding that supported this work. Role, if any, of the funding organisation in the design, implementation, interpretation and reporting. | | Not applicable | |
|  | |  | |

**Bibliography**

1. von Elm E, Altman DG, Egger M, Pocock SJ, Gotzsche PC, Vandenbroucke JP. The Strengthening the Reporting of Observational Studies in Epidemiology (STROBE) Statement: guidelines for reporting observational studies.
2. Ogrinc G, Davies L, Goodman D, Batalden P, Davidoff F, Stevens D. SQUIRE 2.0 (Standards for QUality Improvement Reporting Excellence): revised publication guidelines from a detailed consensus process.
3. Ogrinc G, et al. BMJ Qual Saf 2015;0:1–7. doi:10.1136/bmjqs-2015-004411
