# Supplementary material for: Waste in orthopaedic surgery; an application of the healthcare sustainability mode and effect analysis
Source: Int Orthop. 2025 Aug 8;49(10):2393–401. doi: 10.1007/s00264-025-06629-7 (PMC12488813; doi:10.1007/s00264-025-06629-7)
Supplement: Supplementary file 2 — Supplementary Material 2 [file 264_2025_6629_MOESM2_ESM.docx]

## Supplemental file B

HSMEA steps:

*Definition of the topic*

During this first step the scope of the HSMEA was determined. In our study we included the following procedures: ‘open spinal fusion’ (max. three levels), ‘percutaneous spinal fusion’ (max. three levels), ‘unicompartmental knee arthroplasty’ (cemented UKA), ‘total knee arthroplasty’ (cemented TKA), ‘reverse shoulder arthroplasty’ (uncemented RSA) and’ total hip arthroplasty’ (uncemented THA). All procedures were primary elective surgeries in adults with a degenerative disorder. The patient cohort consisted mainly of elderly individuals, with revision and acute surgeries excluded. Waste collection took place in the operating room. Given the high level of standardisation in these procedures and expected minimal variation within each type of surgery, waste was collected from three patients per procedure.

*Team assembly*

A multidisciplinary team was assembled (step 2) and comprised one sustainability researcher (ICK), one orthopaedic surgeon (OLH), two surgery assistants, one Sterile Processing Specialist, two sustainability employees and two employees from the procurement department. This team decided which procedures and types of waste should be included, drew the process flow chart and the used steps of the decision tree to review the possible sustainability strategies.

*Flowchart creation*

In step three, a flowchart was created to visualise the process. The waste and its disposal method were categorised in eight groups; Polyethylene terephthalate (PETE), polypropylene (PP), other plastics, paper, cardboard, and non-recyclable waste (Figure 2). The category ‘other plastics’ included High-density polyethylene (HDPE), polyvinyl chloride (PVC), low-density polyethylene (LDPE) and polystyrene (PS). The category non-recyclable waste encompassed waste not included in other specified waste categories that were recyclable. All waste was collected from the waste bags and the paper waste boxes in the operating room after the operation. For safety reasons, clinical hazardous waste (including sharps like needles) and glass were collected separately and excluded. IV bags were emptied before weighing, as saline could be flushed into the sewage.

*Hazard analysis*

In the HSMEA each identified sub-step in the flowchart received an environmental hazard score (1). The sub-steps were scored by multiplying the waste weight (kg) with conversion factors (kg CO_2_ equivalents). All data were analysed using Excel (Microsoft office 2016).

*Action and outcome measures*

For all identified substeps alternative waste streams were considered by the interdisciplinary team to reduce the carbon footprint. The impact of these waste streams was compared per substep. The different strategies of the 6R methodology were considered by the multidisciplinary team. The most sustainable strategy was chosen, provided its implementation was feasible in our hospital (table 1). The actions were implemented by the multidisciplinary team.

**Table 1.** Actions that have followed from the HSMEA meeting.

|  | **Action** | **Possible reduction of number of items or grams per operation** |
| --- | --- | --- |
| **General** |  |  |
| All PP | Recycle | 3.667 gr |
| All PETE Plastics | Recycle | 315 gr |
| All Other Plastics | Recycle | 1.079 gr |
| All Paper | Recycle | 285 gr |
| Aluminum Packaging | Recycle | 8 gr |
| Unused Surgical Gloves | Reuse | If 2 gloves |
| Reusable Surgical Gowns | Rethink | 3.22 |
| Syringes | Refrain from action |  |
| Water Hoses | Refrain from action |  |
| Sodium Chloride Bags | Refrain from action |  |
| Bandage And Cotton Wraps | Refrain from action |  |
| **Reverse Shoulder** |  |  |
| Covering Material Table Shoulder 180x180 Cm | Refuse | 1 |
| Extension Hose | Refuse | 1 |
| 50 Ml Syringes | Refuse | 2 |
| Surgical Gown | Reduce | 1 |
| Packaging Trays | Rethink | 1744 gr |
| Patient Covering Drape | Rethink | 1 |
| **Unicompartmental Knee** |  |  |
| Paper Towels | Refuse | 2 |
| Pulse lavage | Refuse | 1 |
| Cement Set (Incl. A Big Syringe, Cement Powder) | Refuse | Various items |
| Adhesive Strip | Refuse | 1 |
| 100 Ml Syringe | Refuse | 1 |
| Surgical Gown | Reduce | 1 |
| Packaging Trays | Rethink | 1319 |
| **Total Knee** |  |  |
| Paper Towels | Refuse | 2 |
| Surgical Gown | Rethink | 1 |
| 100 Ml Syringe | Refuse | 1 |
| Adhesive strip | Refuse | 1 |
| Crepe Paper Packaging Trays | Rethink | 1631 |
| **Total Hip** |  |  |
| Patient Covering Drape | Rethink | 377 |
| Surgical Gown | Reduce | 1 |
| Instruction Manual | Rethink | 2 |
| Packaging Trays | Rethink | 1051 gr |
| **Open Spinal Fusion** |  |  |
| Packaging Trays | Rethink | 1889 gr |
| The Lamp Covers | Refrain from action |  |
| **Percutaneous Spinal Fusion** |  |  |
| Packaging Trays | Rethink | 1846 gr |
| The Lamp Covers | Refrain from action |  |

This article used CO_2_-eq. values for material use, recycling and incineration from one database and two reports (Table 2). The choice was made to use open public sources; they allow to share the used CO^2^-eq. values. The sources used, in order of preference, were: Idemat 2024-V2-1 2024 (2), Wrap 2010 (3), and Defra 2011(4). The Idemat database was given the highest preference because it is the most recent publication. Wrap, which advises Defra on waste reduction, was preferred over Defra as the Wrap 2010 report contains newer information than the Defra 2011 paper. The values that were chosen from the different databases can be found in Table 3, 4 and 5.

**Table 2.** Overview of the used conversion factors (kg CO^2-^eq./ kg)

|  | **Material use** | **Recycling(upcycling credit)** | **Incineration** |
| --- | --- | --- | --- |
| PETE plastic | 2.051337744 | -0.926645489 | 2.29 |
| Propylene | 1.63 | 0.246035782 | 1.227009474 |
| Other plastics | 3.179 | 0.03 | 1.829 |
| Paper | 0.376390053 | -0.78 | -0.17 |
| Cardboard | 0.419747308 | 0.1831 | 0.64278 |
| Aluminium | 9.844 | -9.245 | 0.031 |
| Mixed non-recyclable waste | 2.053 | / | 0.037 |

**Table 3.** Information from Idemat 2024-V2-1 2024 database, CO_2_^-^eq. emission factors (kg CO^2-^eq./ kg)

|  |  |  | Unit |  | Carbon footprint kg CO2 equiv. |
| --- | --- | --- | --- | --- | --- |
| A.130.07.117.230701 PET amorphous | plastics | Materials, plastics, Thermoplasts | kg | PET amorphous | 2.05134 |
| F.120.01.111.230701 PET (Polyethylene terephthalate, closed loop chemical upcycling credit | end-of-life | waste treatment, upcycling credit plastics | kg | PET (Polyethylene terephthalate, closed loop chemical upcycling credit | -0.92665 |
| F.106.03.111.230701 PET (Polyethylene terephthalate) waste combustion, clean tech, without heat recovery | end-of-life | no heat recovery, waste combustion, thermoplasts | kg | PET (Polyethylene terephthalate) waste combustion, clean tech, without heat recovery | 2.29000 |
| A.130.07.121.230701 PP (PP) | plastics | Materials, plastics, Thermoplasts | kg | PP (PP) | 1.63000 |
| F.090.01.116.230701 PP (PP) waste incineration with electricity | end-of-life | waste treatment, non-recyclable waste incineration with electricity, thermoplastics | kg | PP (PP) waste incineration with electricity | 1.22701 |
| F.120.01.116.230701 PP (PP), closed loop chemical upcycling credit | end-of-life | waste treatment, upcycling credit plastics | kg | PP (PP), closed loop chemical upcycling credit | 0.24604 |
| A.120.01.108.230701 Paper, woodfree uncoated, bleached, waste wood based | paper and packaging | Materials, paper and packaging, general | kg | Paper, woodfree uncoated, bleached, waste wood based | 0.37639 |
| A.120.01.111.240310 Folding Boxboard GC1 | paper and packaging | Materials, paper and packaging, general | kg | Folding Boxboard GC1 | 0.41975 |

**Table 4.** Information from Wrap final report Environmental benefits of recycling – 2010 update. CO_2_-eq. emission factors ( kg CO2-eq./ kg)

| Title | Year | Author | Methodology | Included stages | Functional unit | Name | Climate change ( kg CO2 eq/ ton) |
| --- | --- | --- | --- | --- | --- | --- | --- |
| LCA of management strategies for mixed waste  Plastics. | 2008 | Shonfield | CML 2011 | Disposal stage only | 1000 kg of mixed plastic wastes arising from a  materials recycling facility | Incineration with energy  recovery | 1829 |
| LCA of management strategies for mixed waste  Plastics. | 2008 | Shonfield | CML 2011 | Disposal stage only | 1000 kg of mixed plastic wastes arising from a  materials recycling facility | Pyrolysis of PP and PE (+  recycling for PET and PVC) | 30 |
| Solid waste management and greenhouse gases: A life cycle  assessment of emissions and sinks | 2006 | US EPA | IPCC | Disposal stage only | 1 short ton of material office paper | Incineration | -170 |
| Solid waste management and greenhouse gases: A life cycle  assessment of emissions and sinks | 2006 | US EPA | IPCC | Disposal stage only | 1 short ton of material office paper | Recycling | -780 |
| Life cycle assessment of energy from solid waste | 2000 | Finnvenden et al | EDIP. USES‐LCA. Ecoindicator 99 | Disposal stage only | Treatment of the amount of the included waste fractions  collected in Sweden during one year | Incineration | 642.78 |
| Life cycle assessment of energy from solid waste | 2000 | Finnvenden et al | EDIP. USES‐LCA. Ecoindicator 99 | Disposal stage only | Treatment of the amount of the included waste fractions  collected in Sweden during one year mixed cardboard. | Recycling | 183.10 |

**Table 5.** Information from Defra 2011 Guidelines to Defra/ DECC’s GHG Conversion Factors for Company Reporting: Methodology Paper for Emission Factors. Table 55: Life-Cycle GHG Conversion Factors for Waste Disposal. Page 65. (kg CO_2_-eq./ kg)

| Waste fraction | Conversion factor name | Net kgCO2e emitted per tonne of waste treated/disposed |
| --- | --- | --- |
| Metal: Aluminium cans and foil (excl forming) | Production  Emissions  (avoidance  excl disposal).  kg CO2e | 9.844 |
| Metal: Aluminium cans and foil (excl forming) | Closed Loop | -9.245 |
| Metal: Aluminium cans and foil (excl forming) | Energy Recovery Combustion | 31 |
| Plastics: Mean plastics | Production  Emissions  (avoidance  excl disposal).  kg CO2e 2 | 3.179 |
| Mixed non-recyclable waste | Production  Emissions  (avoidance  excl disposal).  kg CO2e 2 | 2.053 |
| Mixed non-recyclable waste | Energy Recovery Combustion | -37 |

**Bibliography**

1. de Ridder EF, Friedericy HJ, van der Eijk AC, Dankelman J, Jansen FW. A New Method to Improve the Environmental Sustainability of the Operating Room: Healthcare Sustainability Mode and Effect Analysis (HSMEA). Sustainability. 2022;14(21):13957.

2. Foundation SIM. Idemat 2024-V2-1 2024 [Available from: <https://www.ecocostsvalue.com/EVR/img/Idemat_2024-V2-1f.xlsx>.

3. WRAP. Environmental benefits of recycling – 2010 update 2010 [Project code: SAP097. Available from: <https://pieweb.plasteurope.com/members/pdf/p215802b.PDF>.

4. DEFRA. 2011 [Available from: <https://www.gov.uk/government/publications/2011-guidelines-to-defra-decc-s-greenhouse-gas-conversion-factors-for-company-reporting-methodology-paper-for-emission-factors>.
